# Safety evaluation of lotilaner in dogs after oral administration as flavoured chewable tablets (Credelio™)

**DOI:** 10.1186/s13071-017-2468-y

**Published:** 2017-11-01

**Authors:** Emmanuelle A. Kuntz, Srinivas Kammanadiminti

**Affiliations:** 1 Elanco Animal Health, Schwarzwaldallee 215, WRO-1032.2.52, CH-4058 Basel, Switzerland; 2 Elanco Animal Health, 2500 Innovation Way, Greenfield, IN 46140 USA

**Keywords:** Lotilaner, Credelio™, Safety, Dog, Oral

## Abstract

**Background:**

Lotilaner (Credelio™, Elanco) is a novel isoxazoline that provides rapid speed of flea and tick knockdown which is sustained for at least 1 month following oral administration to dogs. The safety of lotilaner flavoured chewable tablets was investigated in a randomized, blinded, parallel-group design study in healthy Beagle puppies starting at 8 weeks of age. Lotilaner was administered orally once a month over 8 months at one, three and five times the upper level of the recommended dose range (of 20 to 43 mg/kg).

**Methods:**

The objective of this study was to determine the safety of lotilaner flavoured chewable tablets in healthy dogs when administered monthly over an extended time period at the highest recommended dose rate, i.e. 1× and at elevated dose rates, i.e. 3× and 5×. Sixteen male and 16 female healthy 8-week-old puppies, weighing ~1.5 to 3.0 kg, were randomized among four groups to be untreated controls or to receive lotilaner at dose rates of 43 mg/kg (1×), 129 mg/kg (3×), or 215 mg/kg (5×) on eight occasions - every 4 weeks over 8 months. The control group was sham-dosed. Study dogs were fed within 30 min prior to treatment. Assessment of safety was based on general health observations, detailed clinical observations, complete physical/neurological examinations, including ophthalmological examinations and clinical pathology evaluations (haematology, clinical chemistry and urinalysis), food and water consumption, body weight, pharmacokinetic blood collections, macroscopic and microscopic examinations.

**Results:**

Blood concentrations of lotilaner confirmed systemic exposure of all study dogs with the exception of the control group. Lotilaner did not induce any treatment-related effects on body weight, food consumption, opthalmoscopic, physical/neurological and electrocardiographic examinations. For clinical pathology, no changes related to treatment were noted. There were no treatment-related changes in gross examinations. After microscopic examinations, minor findings recorded in kidneys were of no toxicological relevance. Changes in the reproductive tissues were attributed to the peri-pubertal age and growth of the animals.

**Conclusions:**

Lotilaner was well-tolerated in healthy puppies at 8 week of age when administered once monthly on eight occasion over 8 months at the highest recommended dose and at three and five-fold overdose.

**Electronic supplementary material:**

The online version of this article (10.1186/s13071-017-2468-y) contains supplementary material, which is available to authorized users.

## Background

Lotilaner is a novel isoxazoline that has recently been approved for use in dogs for the rapid and sustained elimination of flea and tick infestations. Members of the isoxazoline class have been shown to kill insects and acari by interfering with neuromuscular and central nervous neurotransmission through binding to receptors that activate ligand-gated chloride channels (γ-aminobutyric acid- and glutamate-gated chloride channels) [1–3]. The safety of these compounds in mammals is due to their significant selectivity for neurons that are present throughout the insect central nervous and neuromuscular systems [3].

Formulated as a flavoured chewable tablet, in recently fed dogs lotilaner (Credelio™) is rapidly absorbed, achieving peak blood concentrations within 2 h after treatment [4]. Lotilaner has a half-life of approximately 30 days, so that insecticidal and acaricidal blood levels are sustained for at least 1 month following treatment [4]. Laboratory studies have indicated that lotilaner will be a valuable drug for veterinarians and dog owners in the management of flea and tick infestations, but before wide-scale use could be recommended, it was important to demonstrate safety in the target canine population following repeated administrations of the highest recommended dose rate [5–7].

A Target Animal Safety study was initiated with the objective of evaluating the safety of lotilaner flavoured chewable tablets in 8-week old Beagle dogs when administered orally as tablets, once every 4 weeks for 8 months. The recommended (minimum) monthly dose rate of lotilaner is 20 mg/kg. Since the tablets are recommended for a weight band, the dose rate range is 20–43 mg/kg to be administered once a month orally. Study treatments targeted achieving multiples of one (1×; 43 mg/kg), three (3×; 129 mg/kg) and five times (5×; 215 mg/kg) the upper level of this dose band.

## Methods

This randomized, controlled, blinded study was conducted with reference to the guidelines for evaluating the target animal safety of new pharmaceuticals [VICH Guideline 43, and to recognized quality assurance standards (United States Food and Drug Administration (FDA) Good Laboratory Practice (GLP) Regulations, 21 Code of Federal Regulations (CFR) Part 58 and the Organization for Economic Cooperation and Development (OECD) Series on Principles of Good Laboratory Practice and Compliance Monitoring, Number 13)] [8–10]. The study was reviewed and approved by the site Ethics Committee and the sponsor company Institutional Animal Care and Use Committee. This manuscript was prepared in compliance with the ARRIVE Guidelines Checklist for animal in vivo experiments [11].

### Animal management

Thirty-two out of 48-week-old Beagle dogs (16 males and 16 females, weighing 1.6 kg to 3.0 kg and 1.5 kg to 2.1 kg, respectively) were selected and acclimatized to the controlled indoor environment for 2 weeks prior to baseline data collection. Animals had not previously been involved in any other experimental study. Beginning on Day -1 and for the duration of the study until the end of the in-life phase, dogs were housed individually in stainless steel mobile cages with plastic-coated flooring. Dry (Lab Diet® Certified Canine Diet #5007, PMI Nutrition International, Inc.) and moistened food (Eukanuba Performance diet) were available ad libitum to all animals from arrival until they were 10 weeks of age, with the exception limited periods prior to administration. During these limited periods, dogs were fasted for six to 12 h and then 30 min prior to treatment. On treatment days, all animals were offered 60 to 80 g of canned food (Hill’s Science Diet A/D or Purina Veterinary Diet DM) along with the ration of moistened Eukanuba diet (Day 1 only) and dry Lab Diet® within 30 min prior to dosing. On Day 141, the canned Hill’s Science Diet A/D was replaced for the remainder of the study with Purina Veterinary Diet DM. Drinking water was available *ad libitum*.

### Randomisation, blinding and treatment

Each animal was randomly allocated on Day -1 to one of the treatment groups based on homogenous distribution of body weight and sex criteria (4 males and 4 females per group) (Table 1). The four groups were: Group 1: Untreated control dogs (sham-dosed with 5 ml of tap water); Group 2: Dogs were treated with lotilaner flavoured chewable tablets at a target dose level of 43 mg/kg (1×); Group 3: Lotilaner flavoured chewable tablets at a target dose level of 129 mg/kg (3×); Group 4: Lotilaner flavoured chewable tablets at a target dose level of 215 mg/kg (5×).

**Table 1:** Range of lotilaner dose rates administered to each of the study groups

	Day of dosing							
	1	29	57	85	113	141	169	197
43 mg/kg (1×)								
Male	44.3–59.5	48.2–57.7	43.3–46.9	43.3–46.5	42.5–45.9	39.5–46.6	40.2–45.3	42.3–44.4
Female	54.1–65.0	44.1–70.3	43.8–51.9	41.2–54.4	40.7–47.9	43.3–46.1	40.9–45.9	45.6–47.5
129 mg/kg (3×)								
Male	123.4–137.8	123.6–130.4	123.6–130.8	128.0–133.5	125.8–132.8	125.8–132.0	129.3–132.7	129.0–131.4
Female	131.6–151.0	119.7–144.2	125.0–136.4	131.6–137.2	126.1–130.2	125.0–132.4	127.2–132.9	129.5–136.1
215 mg/kg (5×)								
Male	209.3–226.3	214.3–225.0	206.3–220.1	218.0–228.0	211.5–218.8	213.0–217.5	214.3–220.1	216.6–220.4
Female	203.0–220.6	209.3–225.0	208.3–220.6	214.3–227.3	211.6–220.1	209.6–212.8	213.5–219.7	217.0–220.9

All personnel involved in recording animal data were blinded to the treatment group allocations and were not involved in administration of treatments. Histopathological evaluation was conducted unblinded.

### Test article administration

For this safety study, the upper end of the dosage range was selected for the 1× dosage, i.e. 43 mg/kg. Doses for each dog were calculated from body weight measured at baseline in the acclimation phase, and during the experimental phase. Tablets (commercial tablets size, not scorable) were provided with lotilaner amounts of 56.25, 112.5, 225 and 450 mg. Single tablets or multiple tablets were administered to achieve as close as possible to the individual target dose. As food has been shown to increase lotilaner absorption dogs were fed within 30 min prior to each dosing [4].

Beginning on Day 0, tablets were administered per os once every 4 weeks for 8 months (Days 1, 29, 57, 85, 113, 141, 169 and 197). A small amount of water was then given and the mouth checked to ensure the tablets had been swallowed. The control animals were sham-treated with 5 ml of tap water.

### General health observations

The general health of all dogs was checked and recorded by an animal technician twice daily, generally 6 h apart. Observations assessed included morbidity, mortality, injury, and the availability of food and water.

### Detailed clinical observations, ophthalmoscopic and electrocardiographic examinations

A detailed clinical examination of each dog was performed on Days -15, -4, -1, then at 8 h (± 1 h) post-dose on each dosing day, and once weekly thereafter, and on Day 225. Observations included, but were not limited to, evaluation of skin, hair coat, eyes, ears, nose, oral cavity, thorax, abdomen, external genitalia, limbs and feet, respiratory and circulatory signs, autonomic effects such as salivation, and nervous system effects including tremors, convulsions, reactivity to handling, and unusual behavior.

Electrocardiographic (ECG) recordings were completed on Day -8, and on Days 59, 143, 199 and 222. The ECG traces from each animal were examined by a certified veterinary cardiologist for the following variables: heart rate, R-R interval, P-R interval, Q-T intervals, QRS duration. Corrected QT (QTc) interval was calculated using a published procedure [12].

On Days -6, 99 and 211, ophthalmoscopic examinations were carried out.

### Body weights and food consumption

Body weights for all animals were measured during the acclimation phase and at least once a week during the study. Food consumption (dry and wet food) was measured and recorded daily.

### Physical/neurological examinations

Complete physical and neurological examinations were conducted on Days -7, 5, 35, 63, 91, 119, 147, 175, 203 and 224. Assessments of toxicity and health included general condition and behavior, general ocular without ophthalmoscope; integument; musculoskeletal; gastrointestinal; body temperature; cardiovascular and respiratory including assessment by auscultation; and reproductive system; lymphatic, urinary and nervous systems. The neurological assessment included observation for nystagmus, pupillary response, extensor thrust (muscle tone), righting reflex, startle reflex, proprioception, and walking movement.

### Clinical pathology

Blood samples for the determination of hematology, clinical chemistry and coagulation variables were collected on all animals at pre-test Day -6 (Day -9 for urinalysis), and at Days 8, 29, 36, 57, 64, 85, 92, 113, 120, 141, 148, 169, 176, 197, 204 and 223. Urine samples were collected using steel pans placed under the cages for at least 16 h. Urinalysis (morphological, microscopic and biochemical) was carried out. The hematology profile included: erythrocytes, hemoglobin, hematocrit, mean corpuscular hemoglobin, mean corpuscular volume, mean corpuscular hemoglobin concentration (calculated), leukocyte count (total and differential), white blood cell differentials (absolute count), platelet count, and absolute reticulocytes. The clinical chemistry profile included: alkaline phosphatase, total bilirubin (with direct bilirubin if total bilirubin exceeds 1 mg/dl), aspartate aminotransferase, alanine aminotransferase, gamma glutamyl transferase, urea nitrogen, creatinine, total protein, albumin, globulin and albumin/globulin ratio (calculated), glucose, total cholesterol, triglycerides, electrolytes (potassium, chloride, sodium) calcium, and phosphorus. The coagulation profile included activated partial thromboplastin time, prothrombin time, and fibrinogen. Urinalysis included determination of bilirubin, urobilinogen, ketones, nitrite, glucose, microscopy of centrifuged sediment, specific gravity, pH, protein and glucose.

### Whole blood and pharmacokinetic analysis

For pharmacokinetic investigations, blood samples were collected from all animals via the jugular vein pre-dose (Day -1) and at six and 24 h post-dose on Days 1 and 113; pre-dose and 24 h post-dose on Days 29, 57, 85, 141, 169 and 197; and on Days 4, 8, 15, 22, 116, 120, 127, 134, 200, 204, 211, 218 and 225. The samples were analyzed for determination of lotilaner concentrations using a validated method by HPLC-MS/MS [4]. The pharmacokinetic parameters were calculated from the individual concentration vs time profiles via non-compartmental analysis. The pharmacokinetic parameters included peak values (Cmax), terminal half-life (T_1/2_), area under the curve (AUC), and accumulation ratio.

### Gross and microscopic evaluations

At the end of the study, the dogs were humanely euthanized by an intravenous injection of sodium pentobarbital solution followed by exsanguination via transection of the femoral vessels. Complete and detailed gross and microscopic examinations were carried out on all animals according to VICH GL 43 under the supervision of a veterinary pathologists [8].

### Statistical methods

All data were analyzed with the statistical software package SAS/STAT® (Version 13.2, Version 9.4 of the SAS System for Windows, Copyright© 2002–2012 by SAS Institute Inc., Cary, NC, USA). The following endpoints were analyzed: organ weights, body weight, ECG variables, clinical pathology (haematology, coagulation, clinical chemistry, urinalysis), dry and wet food consumption, and pharmacokinetic parameters. Each treated group was analyzed compared to the control group.

Endpoints measured once post-treatment that did not include a pre-treatment measurement (e.g. organ weight) were analyzed using analysis of variance (ANOVA) with ‘treatment’, ‘sex’, and ‘treatment by sex’ as fixed effects [13]. Endpoints measured multiple times post-treatment that include a pre-treatment measurement were analyzed using repeated measures analysis of covariance (RMANCOVA) with ‘treatment’, ‘time’, and ‘sex’ and associated two- and three-way interactions; and a covariate all as fixed effects [14]. The pre-treatment value closest to dosing was used as the covariate.

Depending on the significance of the interaction terms (*P* ≤ 0.10 level for two-way interactions and *P* ≤ 0.10 for the three-way interaction), treated groups were compared to the control either within each sex (treatment by sex significant), within each time point (treatment by time significant) or main effect only (neither treatment by sex nor treatment by time significant).

### Translations

Spanish translation of the article is available in Additional file 1. French translation of the Abstract is available in Additional file 2.

## Results and discussion

The target dose rate administered to dogs in each of the lotilaner groups was consistent with the planned dosage scheme (Table 1). Blood concentrations of lotilaner confirmed systemic exposure of all treated dogs.

### General health, detailed clinical observations and ophthalmoscopic evaluations

There were no treatment-related adverse findings in general health observations. No clinical signs related to lotilaner administration were noted during the study. Fecal observations such as watery, soft, mucoid or red-discolored feces were seen in all groups including controls. Vomiting was reported only in two dogs in the control group. Further clinical signs included isolated instances of lacrimation and red discoloration of the gingiva in all groups including controls.

There were no treatment-related effects observed during ophthalmoscopic examinations. The ophthalmological findings of corneal edema in one high-dose dog (5×) and chorioretinitis in another high dose treated animal were unrelated to each other and considered to be unrelated to treatment.

### Body weights and food consumption

From Day 42 onwards, body weights were statistically significantly lower only in male dogs given the lowest lotilaner dose (43 mg/kg) in comparison with control males (RMANCOVA, minimum *P* = 0.0288, *t*_(55.5)_ = 2.24, for day 77). However, there were no other significant (*P* > 0.1) effects. Furthermore, for both wet and dry food consumption, there were no statistically significant treatment-related effects. It was therefore concluded that the test article had no significant effect on body weights or food consumption.

### Physical/neurological examinations

No clinically relevant abnormalities attributable to the treatment were detected during scheduled physical/neurological examinations.

### Electrocardiographic evaluations

All ECG readings were qualitatively and quantitatively within normal limits. When absolute group mean values were evaluated statistically and compared to interval-matched control values, the QRS duration of the data pooled for both sexes in the 43 mg/kg dose was longer than the control group at the terminal interval. As the difference was mild and noted only following the low dose, the difference is not considered treatment-related. The QTc interval in males in the 43 mg/kg group was shorter than in control group at the terminal interval when comparing the pooled QTc interval data from all phases of the study. As the difference in the QTc interval was noted in only one sex and following the low dose, the difference is not considered to be related to treatment. There was no effect of oral administration of lotilaner on qualitative or quantitative ECG parameters.

### Clinical pathology

There were no lotilaner-related effects on haematology, plasma chemistry, coagulation profiles, or urinalysis parameters at any dose level. Any statistically significant changes from baseline were not considered meaningful, based on their small magnitude, lack of dose response, maturation and growth of the dogs during the study, and/or relationship to pre-test and expected historical ranges.

### Organ weights and gross and microscopic examinations

There were no lotilaner-related macroscopic findings at terminal necropsy and no toxicologically meaningful organ weight changes in males or females. Any statistically significant differences between any of the treated groups, relative to controls, were not considered toxicologically meaningful because there were no microscopic correlates to the weight changes, no dose–response relationships, and/or opposite effects were present in males and females. Similarly, there were no definitive lotilaner-related microscopic findings. Occasional findings of vasculitis/perivasculitis are consistent with spontaneous/background vasculitis previously described in Beagle dogs [15–17], and microscopic findings in the reproductive tissues were considered to be associated with the maturation and growth of the dogs during the study.

### Pharmacokinetic analysis

**Fig. 1:**
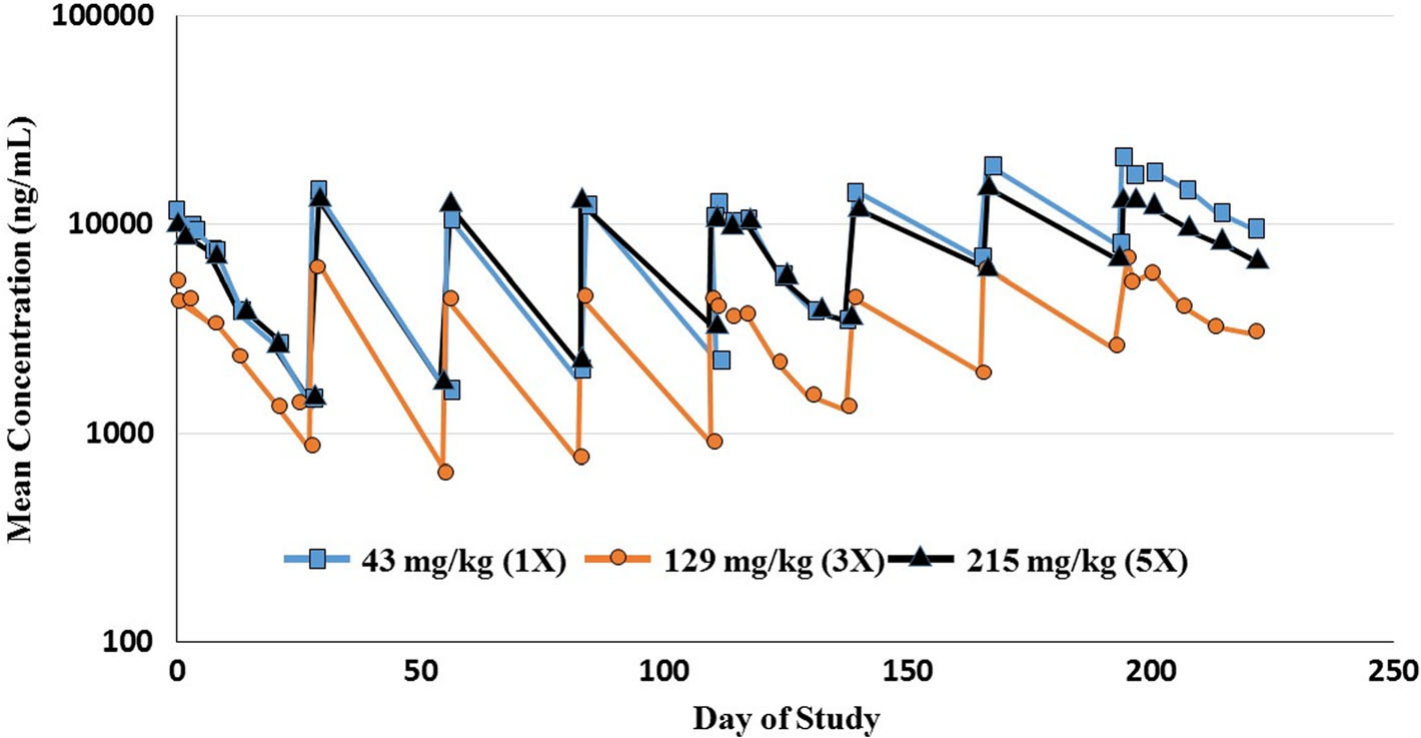
Mean lotilaner whole blood concentration-time profiles following eight consecutive monthly oral administrations of 43 (1×), 129 (3×), and 215 (5×) mg/kg

The low variability of lotilaner in Cmax and AUC_0-672h_ between animals and months throughout the study demonstrates consistent and adequate exposure of all treated dogs. Mean systemic exposure and Cmax values increased with increasing dose in a less than dose proportional manner, especially in the 5× group which was approximately 3-fold instead of 5-fold (Fig. 1). No gender effect was observed. As reported with other isoxazolines, a moderate degree of accumulation (from single treatment to steady state) is expected and for lotilaner can be considered a normal consequence of the relatively long half-life, which in turn provides assurance that efficacy will be sustained over the entire month following treatment [18, 19].

## Conclusions

Careful clinical examinations, clinical pathology assessments and macroscopic/microscopic examinations in this rigorous safety investigation found that eight consecutive monthly lotilaner treatments, at dose rates of up to 215 mg/kg, beginning when puppies were 8 weeks of age did not cause any effects of toxicological concern. The results therefore show that lotilaner flavoured chewable tablets have a wide safety margin when administered at monthly intervals to puppies and dogs, male or female, at the highest dose band rate of 43 mg/kg.

## Additional files

Additional file 1: Spanish translation of the article. (PDF 90 kb)

Additional file 2: French translation of the Abstract. (PDF 35 kb)

## Abbreviations

ANOVA: Analysis of variance
APPT: Activated partial thromboplastin time
ARRIVE: Animal Research Reporting in vitro Experiments
AUC_0-672h_: Mean systemic exposure from 0 to 672 h
CFR: Code of Federal Regulations
Cmax: Peak blood concentration
ECG: Electrocardiograph
FDA: United States Food and Drug Administration
GCP: Good clinical practice
GGT: Gamma-glutamyl transferase
HPLC-MC/MS: High performance liquid chromatography coupled with tandem mass spectrometry
LSMEANS: Least squares means
MCH: Mean corpuscular hemoglobin
OECD: Organization for Economic Cooperation and Development
RMANCOVA: Repeated measures analysis of covariance
SOK: Speed of kill
T_1/2_: Terminal blood half-life
VICH: Veterinary International Conference on Harmonization
