# Supplementary material for: Safety evaluation of lotilaner in dogs after oral administration as flavoured chewable tablets (Credelio™)
Source: Parasit Vectors. 2017 Nov 1;10:538. doi: 10.1186/s13071-017-2468-y (PMC5664904; doi:10.1186/s13071-017-2468-y)
Supplement: Supplementary file 1 — Spanish translation of the article. (PDF 90 kb) [file 13071_2017_2468_MOESM1_ESM.pdf]

Evaluación de seguridad de lotilaner en perros después de la administración oral como tabletas masticables saborizadas (Credelio™)

Emmanuelle A. Kuntz[1]* y Srinivas Kammanadiminti[2]

[1]Elanco Animal Health, Mattenstrasse 24a, WRO-1032.2.52, CH-4058, Basel, Suiza
[2]Elanco Animal Health, 2500 Innovation Way, Greenfield, IN 46140, Estados Unidos

*Correspondencia: emmanuelle.kuntz@elanco.com

E-mails:
EK: emmanuelle.kuntz@elanco.com
SK: srinivas.kammanadiminti@elanco.com

Resumen

Antecedentes: Lotilaner (Credelio™, Elanco) es una isoxazolina nueva que provee una rápida velocidad para eliminar pulgas y garrapatas, la cuál se sostiene por al menos un mes después de la administración oral en perros. La seguridad de las tabletas masticables saborizadas de lotilaner fue investigada en un estudio aleatorizado, ciego con diseño de grupo-paralelo en cachorros Beagle, comenzando a las 8 semanas de edad. Lotilaner fue administrado oralmente una vez al mes por más de ocho meses a una, tres y cinco veces el nivel superior del rango de la dosis recomendada (de 20 a 43 mg/kg).

Métodos: El objetivo de este estudio fue para determinar la seguridad de las tabletas masticables saborizadas de lotilaner en perros sanos cuando se administraron mensualmente en un período extendido de tiempo al nivel de dosis más elevado recomendado, i.e. 1× y a dosis elevadas, i.e. 3× y 5×. Aleatoriamente se dividieron en cuatro grupos dieciséis cachorros machos y 16 hembras sanos de 8 semanas de edad, pesando ~1.5 a 3.0 kg, a recibir lotilaner al nivel de dosis de 43 mg/kg (1×), 129 mg/kg (3×), o 215 mg/kg (5×) en ocho ocasiones – cada cuatro semanas en ocho meses. El grupo control se le dio una dosis simulada. Los perros del estudio fueron alimentados 30 minutos antes del tratamiento. La evaluación de seguridad se basó en las observaciones de salud general, las observaciones clínicas detalladas, los exámenes físico/neurológicos, incluyendo exámenes

oftalmológicos y evaluaciones de patología clínica (hematología, química clínica y urinanálisis), consumo de alimento y agua, peso corporal, farmacocinética en sangre colectada, exámenes macroscópicos y microscópicos.

Resultados: Las concentraciones de sangre de lotilaner confirmaron una exposición sistémica de todos los perros del estudio con excepción del grupo control. Lotilaner no indujo ningún efecto relacionado con el tratamiento sobre el peso corporal, el consumo de alimento, los exámenes oftalmoscópico, físico/neurológico y electrocardiográfico. Para la patología clínica, no se notaron cambios en relación con el tratamiento. No hubo cambios relacionados con el tratamiento en los exámenes macroscópicos. Después de los exámenes microscópicos, se registraron hallazgos menores en los riñones sin relevancia toxicológica. Los cambios en los tejidos reproductivos se atribuyeron a la edad peri-pubertad y al crecimiento de los animales.

Conclusiones: Lotilaner fue bien tolerado en cachorros sanos a las 8 semanas de edad cuando se administró una ves al mes en ocho ocasiones durante ocho meses a la dosis más elevada recomendada y a una sobredosis de tres a cinco veces más.

Palabras clave: Lotilaner, Credelio™, Seguridad, Perro, Oral

Antecedentes

Lotilaner es una isoxazolina nueva que recientemente ha sido aprobada para su uso en perros para una eliminación rápida y sostenida de las infestaciones de pulgas y garrapatas. Se ha demostrado que los miembros de la clase de las isoxazolinas matan insectos y ácaros interfiriendo con la neurotransmisión neuromuscular y central nerviosa a través de la unión a los receptores que activan las compuertas de los ligandos de los canales de cloro (ácido γ-aminobutírico – y canales de cloro con compuerta de glutamato) [1-3]. La seguridad de estos compuestos en mamíferos se debe a su selectividad significativa para las neuronas que están presentes a través de los sistemas nervioso central y neuromuscular de los insectos [3].

Lotilaner (Credelio™) formulado como una tableta masticable saborizada administrada a perros recientemente alimentados se absorbe rápidamente, logrando un pico de concentración en sangre al cabo de dos horas después del tratamiento [4]. Lotilaner tiene una vida media de aproximadamente 30 días, de tal forma que los niveles insecticida y acaricida en sangre se sostienen por al menos un mes después del tratamiento [4]. Los estudios de laboratorio han indicado que lotilaner será un fármaco valioso para los veterinarios y los propietarios de los perros para el manejo

de las infestaciones de pulgas y garrapatas, pero antes de que se recomiende el uso a gran escala, era importante demostrar la seguridad en la población canina blanco después de administraciones repetidas del nivel de dosis más elevado recomendado [5-7].

Se inició un estudio de Seguridad en el Animal Blanco con el objetivo de evaluar la seguridad de las tabletas masticables saborizadas de lotilaner en ocho perros Beagle de ocho semanas de edad cuando se administraron las tabletas oralmente, una vez cada cuatro semanas durante ocho meses. El nivel de dosis (mínimo) recomendado al mes de lotilaner es de 20 mg/kg. Puesto que las tabletas se recomiendan para una banda de peso, el rango del nivel de dosis es de 20–43 mg/kg para ser administrado una vez al mes oralmente. Los tratamientos del estudio fueron dirigidos para lograr múltiplos de uno (1×; 43 mg/kg), de tres (3×; 129 mg/kg) y cinco veces (5×; 215 mg/kg) el nivel superior de la banda de dosis.

Métodos

Este estudio aleatorizado, controlado y ciego fue conducido con referencia a las guías para evaluar la seguridad en el animal blanco de nuevos farmacéuticos [VICH Guía 43, y para reconocer los estándares del aseguramiento de la calidad (Administración de Alimentos y Fármacos de los Estados Unidos (FDA) Regulaciones de Buenas Prácticas de Laboratorio (GLP), Código 21 de Regulaciones Federales (CFR) Parte 58 y la Organización para la Cooperación y Desarrollo Económico (OECD) Series en los Principios de las Buenas Prácticas de Laboratorio y Monitoreo del Cumplimiento, Número 13)] [8-10]. El estudio fue revisado y aprobado por el sitio del Comité Ético y el Comité Institucional del Cuidado y Uso Animal de la compañía patrocinadora. Este manuscrito fue preparado en cumplimiento con la Lista de Guías ARRIVE para experimentos en animales *in vivo* [11].

Manejo animal

Treinta y dos de cuarenta perros Beagle de ocho semanas de edad (16 machos y 16 hembras, pesando de 1.6 kg a 3.0 kg y de 1.5 kg a 2.1 kg, respectivamente) fueron seleccionados y aclimatizados al ambiente controlado interior durante dos semanas previas a la colección de datos en la línea base. Los animales no habían sido involucrados previamente en ningún otro estudio experimental. Comenzando en el Día -1 y para la duración del estudio hasta el final de la fase en-vida, los perros fueron alojados individualmente en jaulas móviles de acero inoxidable con pisos

cubiertos de plástico. Hubo alimento seco (Lab Diet® Certified Canine Diet #5007, PMI Nutrition International, Inc.) y alimento húmedo (Eukanuba Performance diet) disponible *ad libitum* para todos los animales desde su llegada hasta que tuvieron 10 semanas de edad, con excepción de períodos limitados previos a la administración. Durante estos períodos limitados, los perros fueron ayunados durante seis a 12 horas y luego 30 minutos antes de la administración. Durante estos períodos limitados, los perros fueron ayunados durante seis a 12 horas y luego 30 minutos previos al tratamiento. En los días de tratamiento, a todos los animales se les ofrecieron 60 a 80 g de alimento enlatado (Hill's Science Diet A/D o Purina Veterinary Diet DM) junto con la ración de la dieta húmeda de Eukanuba (Día 1, solamente) y la dieta seca Lab Diet® dentro de los 30 minutos previos a la dosificación. En el Día 141, la dieta enlatada de Hill's Science Diet A/D se reemplazó para lo que quedaba del estudio con la dieta de Purina Veterinary Diet DM. El agua de bebida estuvo disponible *ad libitum*.

Aleatorización, cegado y tratamiento

Cada animal fue ubicado aleatoriamente en el Día -1 a uno de los grupos de tratamiento basado en la distribución homogénea del peso corporal y criterio de sexo (4 machos y 4 hembras por grupo) (Tabla 1). Los cuatro grupos fueron: Grupo 1: Perros en el control no tratado (dosis-simulada con 5 ml de agua de la llave); Grupo 2: Perros tratados con tabletas masticables saborizadas a un nivel de dosis blanco de 43 mg/kg (1×); Grupo 3: Tabletas masticables saborizadas de lotilaner a un nivel de dosis objetivo de 129 mg/kg (3×); Grupo 4: Tabletas masticables saborizadas de lotilaner a un nivel de dosis objetivo de 215 mg/kg (5×).

Todo el personal involucrado en el registro de los datos del animal estaban ciegos a las ubicaciones del grupo de tratamiento y no se involucraron en la administración de los tratamientos. La evaluación histopatológica se condujo de manera no ciega.

Administración del artículo de prueba

Para este estudio de seguridad, el extreme superior del rango de dosis se seleccionó para la dosificación de 1×, i.e. 43 mg/kg. Las dosis para cada perro se calcularon a partir del peso corporal medido en la línea base en la fase de aclimatización, y durante la fase experimental. Las tabletas se proporcionaron (tabletas de tamaño comercial, no marcadas) con cantidades de lotilaner de 56.25, 112.5, 225 y 450 mg. Se administraron una o varias tabletas para lograr la dosis individual objetivo lo más cerca posible. Como se ha demostrado el alimento aumenta la absorción de lotilaner, por lo que los perros se alimentaron dentro de los 30 minutos previos a cada dosificación [4].

Comenzando en el Día 0, se administraron las tabletas *per os* una vez cada cuatro semanas durante ocho meses (Días 1, 29, 57, 85, 113, 141, 169 y 197). Una pequeña cantidad de agua se administraba y se revisaba la boca para asegurar que las tabletas habían sido tragadas. Se simuló el tratamiento en los animales control con 5 ml de agua de la llave.

Observaciones de salud general

Un técnico en animales revisó y registró la salud general de todos los perros dos veces al día, generalmente con seis horas de diferencia. Las observaciones evaluadas incluyeron morbilidad, mortalidad, lesiones y la disponibilidad de agua y alimento.

Observaciones clínicas detalladas, exámenes oftalmoscópicos y electrocardiográficos

Un examen clínico detallado de cada perro se llevó a cabo en los Días -15, -4, -1, luego a las 8 horas (±1 hora) post-dosis en cada día de dosificación, y una vez a la semana de ahí en adelante, y hasta el Día 225. Las observaciones incluyeron, más no se limitaron a, la evaluación de la piel, el pelaje, los ojos, las orejas, la nariz, la cavidad oral, el tórax, el abdomen, los genitales externos, los miembros y patas, signos respiratorios y circulatorios, efectos autonómicos tales como la salivación y los efectos del Sistema nervioso incluyendo temblores, convulsiones, reactividad al manejo y comportamiento inusual.

Los registros electrocardiográficos (ECG) se completaron en el Día -8, y en los Días 59, 143, 199 y 222. Los trazos del ECG de cada animal fueron examinados por un cardiólogo veterinario certificado para determinar las siguientes variables: frecuencia cardíaca, intervalo R-R, intervalo P-R, intervalos Q-T, duración QRS. El intervalo QT corregido (QTc) se calculó usando un procedimiento publicado [12].

En los Días -6, 99 y 211, se llevaron a cabo los exámenes oftalmoscópicos.

Pesos corporales y consumo de alimento

Los pesos corporales para todos los animales se midieron durante la fase de aclimatización y al menos una vez a la semana durante el estudio. El consumo de alimento (alimento seco y húmedo) se midió y se registró diariamente.

Exámenes físico/neurológicos

Los exámenes físico y neurológicos completos se condujeron en los Días -7, 5, 35, 63, 91, 119, 147, 175, 203 y 224. Las evaluaciones de toxicidad y salud incluyen la condición general y el

comportamiento, ocular general sin oftalmoscopio; examen tegumentario; musculoesquelético; gastrointestinal; temperatura corporal; cardiovascular y respiratorio incluyendo la evaluación por auscultación; y examen del sistema reproductor; linfático, urinario y nervioso. La evaluación neurológica incluyó la observación de nistagmus, respuesta pupilar, impulso extensor (tono muscular), reflejo para enderezarse, reflejo de sobresalto, propiocepción y movimiento ambulatorio.

Patología clínica

Las muestras de sangre para la determinación de hematología, química clínica y las variables de coagulación se colectaron en todos los animales en la pre-prueba Día -6 (día -9 para urianálisis), y a los Días 8, 29, 36, 57, 64, 85, 92, 113, 120, 141, 148, 169, 176, 197, 204 y 223. Las muestras de orina se colectaron usando charolas de acero colocadas abajo de las jaulas por al menos unas 16 horas. Se llevó a cabo el urinanálisis (morfológico, microscópico y bioquímico). El perfil de hematología incluye: eritrocitos, hemoglobina, hematocrito, media de la hemoglobina corpuscular, media de la concentración de hemoglobina corpuscular (calculada), conteo de leucocitos (total y diferencial), los diferenciales de leucocitos (conteo absoluto), conteo de plaquetas y reticulocitos absoluto. El perfil de la química clínica incluye: la fosfatasa alcalina, bilirrubina total (con bilirrubina directa si la bilirrubina total excede a 1 mg/dl), la aspartato aminotransferasa, la alanina aminotransferasa, gama glutamil transferasa, urea nitrógeno, creatinina, proteína total, albúmina, globulina y la proporción de albúmina/globulina (calculada), glucosa, colesterol total, triglicéridos, electrolitos (potasio, cloro, sodio) calcio y fósforo. El perfil de la coagulación incluye el tiempo parcial activado de tromboplastina, el tiempo de protrombina y fibrinógeno. El urinanálisis incluyó la determinación de la bilirrubina, urobilinógeno, cetonas, nitrito, glucosa, microscopía del sedimento centrifugado, gravedad específica, pH, proteína y glucosa.

Análisis de sangre complete y farmacocinética

Para las investigaciones de farmacocinética, las muestras de sangre se colectaron de todos los animales a partir de la vena yugular pre-dosis (Día -1) y a las seis y 24 horas post-dosis en los Días 1 y 113; pre-dosis y a las 24 horas post-dosis en los Días 29, 57, 85, 141, 169 y 197; y en los Días 4, 8, 15, 22, 116, 120, 127, 134, 200, 204, 211, 218 y 225. Las muestras se analizaron para la determinación de las concentraciones de lotilaner usando un método validado por HPLC-MS/MS [4]. Los parámetros de farmacocinética se calcularon de la concentración individual *vs* los perfiles de tiempo vía análisis no-compartamental. Los parámetros farmacocinéticos incluyen valores pico (Cmax), vida media terminal ($T_{1/2}$), área bajo la curva (AUC), y la proporción de la acumulación.

Evaluaciones macroscópicas y microscópicas

Al final del estudio, a los perros se les realizó eutanasia humanitariamente por la vía de una inyección intravenosa con solución de pentobarbital sódico seguido de su desangrado vía el corte de los vasos femorales. Los exámenes completos y detallados, tanto macroscópica, como microscópicamente se llevaron a cabo en todos los animales de acuerdo con el VICH GL 43 bajo la supervisión de patólogos veterinarios [8].

Métodos estadísticos

Todos los datos se analizaron con un paquete de software estadístico SAS/STAT® (Version 13.2, Version 9.4 del Sistema SAS para Windows, Copyright© 2002-2012 por SAS Institute Inc., Cary, NC, USA). Los siguientes puntos finales se analizaron: pesos de los órganos, peso corporal, variables de ECG, patología clínica (hematología, coagulación, química clínica, urinanálisis), consumo de alimento seco y húmedo, parámetros de farmacocinética. Cada grupo tratado se analizó comparándolo con el grupo control.

Las medidas de los puntos finales realizadas una vez post-tratamiento, no incluyeron una medida pre-tratamiento (e.g. peso del órgano) se analizaron usando el análisis de varianza (ANOVA) con ‘tratamiento’, ‘sexo’, y ‘tratamiento por sexo’ como efectos fijos [13]. Los puntos finales medidos en múltiples ocasiones post-tratamiento que incluyeron una medida pre-tratamiento se analizaron usando medidas repetidas de análisis de covarianza (RMANCOVA) con ‘tratamiento’, ‘tiempo’, y ‘sexo’ y asociadas con interacciones de dos- y tres- vías; y un covariado como efectos fijos [14]. El valor del pre-tratamiento más cercano a la dosificación se utiliza como covariado.

Dependiendo de la significancia de los términos de interacción ($P \leq 0.10$ nivel para interacciones de dos vías y $P \leq 0.10$ para la interacción de tres-vías), los grupos tratados se compararon con el control, ya sea dentro de cada sexo (tratamiento por sexo significativo), dentro de cada punto en el tiempo (tratamiento por tiempo significativo) o solo el efecto principal (ni tratamiento por sexo, ni tratamiento por tiempo significativo).

Resultados y discusión

El nivel de dosis blanco administrado a los perros en cada uno de los grupos de lotilaner fue consistente con el esquema de dosificación planeado (Tabla 1). Las concentraciones de sangre de lotilaner confirmaron la exposición sistémica de todos los perros tratados.

Salud general, observaciones clínicas detalladas y evaluaciones oftalmoscópicas

No hubo hallazgos adversos relacionados con el tratamiento en las observaciones generales de salud. No se notaron signos clínicos relacionados con la administración de lotilaner durante el estudio. Las observaciones fecales, tales como heces acuosas, blandas, mucoides o rojizas-decoloradas fueron vistas en todos los grupos incluyendo a los controles. El vómito se reportó solo en dos perros del grupo control. Otros signos clínicos incluyeron casos de lagrimeo y descoloración roja de la gingiva en todos los grupos incluyendo a los animales control.

No hubo efectos observados relacionados con el tratamiento durante los exámenes oftalmoscópicos. Los hallazgos oftalmoscópicos del edema corneal en un perro que recibió una dosis alta (5x) y la corioretinitis en otro animal tratado con dosis alta no se relacionaron entre sí y se consideró que no estaban relacionados con el tratamiento.

Pesos corporales y consumo de alimento

Del Día 42 en adelante, los pesos corporales fueron más bajos estadísticamente significativos solo en perros machos que recibieron la dosis de lotilaner más baja (43 mg/kg) en comparación con los machos control (RMANCOVA, mínimo $P = 0.0288$, $t_{(55.5)} = 2.24$, para el día 77). Sin embargo, no hubo otros efectos significativos ($P > 0.1$). Más aún, tanto para el consumo del alimento húmedo, como el seco, no hubo efectos estadísticamente significativos relacionados con el tratamiento. Por lo tanto, se concluyó que el artículo de prueba no tuvo efecto significativo en los pesos corporales o en el consume de alimento.

Exámenes físico/neurológicos

No hubo anormalidades clínicamente relevantes atribuibles al tratamiento detectadas durante los exámenes físico/neurológicos programados.

Evaluaciones electrocardiográficas

Todas las lecturas de ECG estuvieron dentro de los límites normales cualitativa y cuantitativamente. Cuando la media de los valores absolutos del grupo se evaluaron estadísticamente y se compararon con los valores control coincidentes con el intervalo, la duración de QRS de los datos agrupados para ambos sexos en la dosis de 43 mg/kg fue mayor que en el grupo control en el intervalo terminal. Como la diferencia fue leve y se notó solo después de la dosis baja, la diferencia no se considera relacionada con el tratamiento. El intervalo QTc en machos en el grupo de 43 mg/kg fue

más corto que en el grupo control en el intervalo terminal cuando se compararon los datos agrupados de QTc de todas las fases del estudio. Como la diferencia en el intervalo QTc se notó solo en un sexo y después de la dosis baja, la diferencia no se consideró que estuviera relacionada con el tratamiento. No hubo efecto de la administración oral de lotilaner sobre los parámetros cualitativos o cuantitativos de ECG.

Patología clínica

No hubo efectos relacionados con lotilaner en hematología, química en plasma, perfiles de coagulación, o parámetros de urianálisis a cualquier nivel de dosis. Cualquier cambio estadísticamente significativo de la línea base no se consideró importante, basándose en la pequeña magnitud, la falta de dosis respuesta, maduración y crecimiento de los perros durante el estudio, /o la relación con la pre-prueba y los rangos históricos esperados.

Los pesos de los órganos y los exámenes macroscópicos y microscópicos

No hubo hallazgos macroscópicos relacionados con lotilaner en la necropsia terminal y no hubo cambios en el peso de órganos significativos toxicológicamente en machos o hembras. Cualquier diferencia estadísticamente significativa entre cualquier grupo tratado, relativo a los controles, no se consideró significativo toxicológicamente porque no hubo correlaciones microscópicas con los cambios de peso, no hubo relaciones dosis-respuesta, y/o efectos opuestos presentes en machos y hembras. De manera similar, no hubo hallazgos microscópicos definitivos relacionados con lotilaner. Los hallazgos ocasionales de vasculitis/perivasculitis son consistentes con la vasculitis espontánea/de fondo previamente descrita en perros Beagle [15-17], y los hallazgos microscópicos en los tejidos reproductivos se consideraron estar asociados con la maduración y el crecimiento de los perros durante el estudio.

Análisis farmacocinético

La baja variabilidad de lotilaner en Cmax y $AUC_{0\text{-}672hr}$ entre los animales y los meses a lo largo del estudio demuestra la exposición consistente y adecuada de todos los perros tratados. La media de los valores sistémicos de la exposición y la Cmax aumentados con una dosis creciente en una manera menos que proporcional a la dosis, especialmente en el grupo 5× que fue de aproximadamente 3-veces en lugar de 5-veces (Fig. 1). No se observó efecto de género. Igual que como se ha reportado con otras isoxazolinas , un grado moderado de acumulación (de un solo tratamiento a un estado estable) es esperado y para lotilaner puede ser considerada una consecuencia normal de la

relativamente larga vida media, que a cambio provee del aseguramiento de que la eficacia será sostenida durante todo el mes después del tratamiento [18, 19].

Conclusiones

Los exámenes clínicos cuidadosos, las evaluaciones de patología clínica y los exámenes macroscópicos/microscópicos en esta rigurosa investigación de seguridad determinaron que ocho tratamientos mensuales consecutivos con lotilaner, a niveles de dosis de hasta 215 mg/kg, comenzando cuando los cachorros tenían ocho semanas de edad y no causaron ningún efecto toxicológico de preocupación. Por lo tanto, los resultados demostraron que las tabletas masticables saborizadas de lotilaner tienen un amplio margen de seguridad cuando se administran en intervalos mensuales a los cachorros y perros, machos o hembras, al nivel de la banda de la dosis más elevada de 43 mg/kg.

Abreviaturas

ANOVA: análisis de varianza; APPT: tiempo de tromboplastina parcial activado; ARRIVE Investigación Animal Reportando Experimentos in vitro; $AUC_{0\text{-}672hr:}$ media de la exposición sistémica de 0 a 672 h; CFR: Código de Regulaciones Federal; Cmax: concentración pico en sangre; ECG: electrocardiógrafo; FDA: Administración de Alimentos y Fármacos de Estados Unidos; GCP: buenas prácticas clínicas; GGT: gama-glutamil transferasa; HPLC-MC/MS: cromatografía líquida de alto desempeño acoplada con espectrometría de masas en tandem; MCH: media de hemoglobina corpuscular; OECD: Organización para la Cooperación Económica y Desarrollo; LSMEANS: medias de mínimos cuadrados; RMANCOVA: medidas repetidas del análisis de covarianza; SOK: velocidad para matar; $T_{1/2}$: vida media terminal en sangre; VICH: Conferencia Internacional de Veterinaria sobre Armonización.

Reconocimientos

Agradecemos al Dr. Bill Ryan de Ryan Mitchell Associates LLC por su asistencia con el manuscrito y al personal de MPI Research, Inc, North Main Street, Mattawan, Michigan, US, incluyendo a Joyce Heward por haber conducido el estudio.

Aprobación de ética

El estudio se basó en las guías para evaluar la seguridad en el animal blanco de nuevos farmacéuticos [VICH Guía 43, y reconocer los estándares de aseguramiento de la calidad (Administración de Alimentos y Fármacos de los Estados Unidos (FDA) Regulaciones de Buenas Prácticas de Laboratorio (GLP), Código 21 de Regulaciones Federales (CFR) Parte 58 y la Organización para la Cooperación y Desarrollo Económico (OECD) Series en los Principios de las Buenas Prácticas de Laboratorio y Monitoreo del Cumplimiento, Número 13)]. El estudio fue revisado y aprobado por el sitio del Comité Ético y el Comité Institucional del Cuidado y Uso Animal de la compañía patrocinadora. Este manuscrito fue preparado en cumplimiento con la Lista de Guías ARRIVE para experimentos en animales *in vivo*.

Consentimiento para publicación

No aplicable.

Disponibilidad de datos y material

Los datos que apoyan las conclusiones de este artículo están incluidas dentro de este artículo.

Intereses competentes

EAK y SK son empleados de Elanco.

Fondos

Todos los estudios fueron patrocinados por Elanco.

Contribuciones de los autores

EAK realizó el preliminar del manuscrito y todos los autores revisaron y aprobaron la versión final. Todos los autores participaron en el diseño del estudio, en la interpretación de los resultados y en la completaron el estudio. Todos los autores leyeron y aprobaron el manuscrito final.

Referencias

1. Garcia-Reynaga P, Zhao C, Sarpong R, Casida JE. New GABA/glutamate receptor target for [(3)H]isoxazoline insecticide. Chem Res Toxicol. 2013;26:514–6.

2. Gassel M, Wolf C, Noack S, Williams H, Ilg T. The novel isoxazoline ectoparasiticide fluralaner: selective inhibition of arthropod γ-aminobutyric acid- and L-glutamate-gated chloride channels and insecticidal/acaricidal activity. Insect Biochem Mol Biol. 2014;45:111–24.
3. Ozoe Y, Asahi M, Ozoe, F, Nakahira K, Mita T. The antiparasitic isoxazoline A1443 is a potent blocker of insect ligand-gated chloride channels. Biochem Biophys Res Commun. 2010;391;744-9.
4. Toutain CE, Seewald W, Jung M. The intravenous and oral pharmacokinetics of lotilaner and the effect of food in dogs. Parasit Vectors (In press).
5. Cavalleri D, Murphy M, Seewald W, Drake J, Nanchen S. Assessment of the speed of flea kill of lotilaner (Credelio™) throughout the month following oral administration to dogs. Parasit Vectors (In press).
6. Murphy M, Garcia R, Karadzovska D, Cavalleri D, Snyder DE, Seewald W et al. Laboratory evaluations of the immediate and sustained effectiveness of lotilaner (Credelio™) against four common species of ticks affecting dogs in North America. Parasit Vectors (In press).
7. Murphy M, Cavalleri D, Seewald W, Drake J, Nanchen S. Laboratory evaluation of the speed of kill of lotilaner (Credelio™) against *Ixodes ricinus* ticks on dogs. Parasit Vectors (In press).
8. VICH GL 43: Guideline on target animal safety for veterinary pharmaceutical products. Belgium; 2008. http://www.ema.europa.eu/docs/en_GB/document_library/Scientific_guideline/2009/10/WC500004361.pdf. Accessed February 12, 2017.
9. United States Food and Drug Administration. Good laboratory practices (GLP) for non-clinical laboratory studies 21 CFR Part 58. 2016. http://www.fda.gov/ohrms/dockets/98fr/980335s1.PDF. Accessed February 12, 2017.
10. OECD series on principles of good laboratory practice (GLP) and compliance monitoring. 1998. http://www.oecd.org/chemicalsafety/testing/oecdseriesonprinciplesofgoodlaboratorypracticeglpandcompliancemonitoring.htm. Accessed February 12, 2017.
11. Kilkenny C, Browne WJ, Cuthill IC, Emerson M, Altman DG. Improving bioscience research reporting: The ARRIVE guidelines for reporting animal research. J Pharmacol Pharmacother. 2010;1:94-9.
12. Fridericia LS. Die Systolendauer im Elektrokardiogramm bei normalen Menschen und bei Herzkranken. Acta Med Scand. 1920;53:469-86.
13. Snedecor, GW, Cochran, WG. Statistical Methods, Eighth edition. Ames (IA): Iowa State Press; 1989.

14. Milliken GA, Johnson DE. Analysis of Messy Data Volume III: Analysis of Covariance. London: Chapman and Hall: 2002.
15. Harcourt RA. Polyarteritis in a colony of beagles. Vet Rec. 1978;102:519-22.
16. Hayes TJ, Roberts GKS, Halliwell WH. An idiopathic febrile necrotizing arteritis syndrome in the dog: beagle pain syndrome. Toxicol Pathol. 1989;17:129-37.
17. Ruben Z, Deslex P, Nash G, Redmond NI, Poncet M, Dodd DC. Spontaneous disseminated panarteritis in laboratory beagle dogs in a toxicity study: a possible genetic predilection. Toxicol Pathol. 1989;17;145-52.
18. Drag M, Saik J, Harriman J, Larsen D. Safety evaluation of orally administered afoxolaner in 8-week-old dogs. Vet Parasitol. 2014;201:198-203.
19. United States Food and Drug Administration. Simparica Prescribing Information. 2016. URL: http://www.accessdata.fda.gov/spl/data/1298a2b8-2424-4b14-a95e-3256a8b5b12b/1298a2b8-2424-4b14-a95e-3256a8b5b12b.xml. Accessed February 12, 2017.

Figura leyenda

Fig. 1 Media de los perfiles de concentración-tiempo de lotilaner en sangre completa después de ocho administraciones orales mensuales consecutivas de 43 (1×), 129 (3×), y 215 (5×) mg/kg

Tabla 1 Rango de los niveles de dosis de lotilaner administrada a cada uno de los grupos de estudio

	**Día de dosificación**							
	**1**	**29**	**57**	**85**	**113**	**141**	**169**	**197**
43 mg/kg (1×)								
Macho	44.3–59.5	48.2–57.7	43.3–46.9	43.3–46.5	42.5–45.9	39.5–46.6	40.2–45.3	42.3–44.4
Hembra	54.1–65.0	44.1–70.3	43.8–51.9	41.2–54.4	40.7–47.9	43.3–46.1	40.9–45.9	45.6–47.5
129 mg/kg (3×)								
Macho	123.4–137.8	123.6–130.4	123.6–130.8	128.0–133.5	125.8–132.8	125.8–132.0	129.3–132.7	129.0–131.4
Hembra	131.6–151.0	119.7–144.2	125.0–136.4	131.6–137.2	126.1–130.2	125.0–132.4	127.2–132.9	129.5–136.1
215 mg/kg (5×)								
Macho	209.3–226.3	214.3–225.0	206.3–220.1	218.0–228.0	211.5–218.8	213.0–217.5	214.3–220.1	216.6–220.4
Hembra	203.0–220.6	209.3–225.0	208.3–220.6	214.3–227.3	211.6–220.1	209.6–212.8	213.5–219.7	217.0–220.9
