# Supplementary material for: Safety evaluation of lotilaner in dogs after oral administration as flavoured chewable tablets (Credelio™)
Source: Parasit Vectors. 2017 Nov 1;10:538. doi: 10.1186/s13071-017-2468-y (PMC5664904; doi:10.1186/s13071-017-2468-y)
Supplement: Supplementary file 2 — French translation of the Abstract. (PDF 35 kb) [file 13071_2017_2468_MOESM2_ESM.pdf]

Évaluation de l'innocuité du lotilaner chez le chien après administration par voie orale sous forme de comprimés à mâcher aromatisés (Credelio™).

Emmanuelle A. Kuntz[1]* et Srinivas Kammanadiminti[2].

[1]Elanco Santé animale, Mattenstrasse 24a, CH-4058 Bâle, WRO-1032.2.58, Suisse

[2]Elanco Santé animale, 2500 Innovation Way, Greenfield, IN 46140, États-Unis

*Correspondance : emmanuelle.kuntz@elanco.com

Adresse électronique :

emmanuelle.kuntz@elanco.com

srinivas.kammanadiminti@elanco.com

Résumé

Contexte : Le lotilaner (Credelio™, Elanco) est un nouvel agent de la classe des isoxazolines agissant rapidement contre les infestations par les puces et les tiques, ayant une activité persistante pendant au moins un mois après une administration orale chez le chien. L'innocuité des comprimés à mâcher aromatisés à base de lotilaner a été explorée lors d'une étude randomisée, en groupes parallèles, en aveugle menée chez des chiots sains de race Beagle âgés de huit mois. Le lotilaner a été administré par voie orale, une fois par mois pendant huit mois à des doses correspondant à une, trois et cinq fois la dose maximale recommandée (20 à 43 mg/kg).

Méthodes : L'objectif de cette étude était de déterminer l'innocuité des comprimés à mâcher aromatisés à base de lotilaner chez des chiens sains lors d'administration mensuelle pendant une période prolongée à la dose maximale recommandée (c-à-d. 1x) et à des doses supérieures (c.-à-d. 3x et 5x). Seize chiots mâles et 16 femelles âgés de huit mois, pesant ~1,5 à 3,0 kg, ont été répartis de façon aléatoire dans quatre groupes : contrôles (non traités) ou traités recevant le lotilaner à des doses de 43 mg/kg (1×), 129 mg/kg (3×) ou 215 mg/kg (5×) à huit reprises, toutes les quatre semaines pendant huit mois. L'administration des comprimés a été simulée dans le groupe contrôle. Les chiens de l'étude ont été nourris dans les 30 minutes précédant le traitement. L'innocuité a été évaluée basée sur des observation de l'état clinique général, d’observations cliniques détaillées, d’examens cliniques/neurologiques complets, incluant des examens ophtalmologiques et des analyses biologiques (hématologie, biochimie clinique et analyse d'urine), la consommation d'eau et

de nourriture, du poids corporel, de prélèvements sanguins destinés aux analyses pharmacocinétiques et des examens macroscopiques et microscopiques.

Résultats : Les concentrations sanguines de lotilaner ont confirmé l'exposition systémique de tous les chiens de l'étude, à l'exception de ceux du groupe contrôle. Le lotilaner n'a induit aucun effet lié au traitement sur le poids, la consommation alimentaire, les examens ophtalmoscopique et clinique/neurologique et l'électrocardiogramme. Il n'a été observé aucune variation des paramètres biologiques liée au traitement. Aucune modification liée au traitement n'a été observée lors des examens cliniques généraux. L'examen microscopique a révélé des modifications mineures au niveau des reins, sans signification toxicologique. Les modifications des tissus reproducteurs ont été attribuées au statut péri-pubertaire et à la croissance des animaux.

Conclusions : Le lotilaner est bien toléré chez les chiots sains âgés de huit semaines, après avoir été administré une fois par mois pendant huit mois à la dose maximale recommandée, ainsi qu’à des multiples, c.-à-d. trois et cinq fois, de la dose maximale recommandée.
